# Chinese Visceral Adiposity Index Is More Closely Associated With Hypertension and Prehypertension Than Traditional Adiposity Indices in Chinese Population: Results From the REACTION Study

**DOI:** 10.3389/fendo.2022.921997

**Published:** 2022-06-30

**Authors:** Binqi Li, Jie Wang, Xin Zhou, Yang Liu, Weiqing Wang, Zhengnan Gao, Xulei Tang, Li Yan, Qin Wan, Zuojie Luo, Guijun Qin, Lulu Chen, Guang Ning, Yiming Mu

**Affiliations:** ^1^ School of Medicine, Nankai University, Tianjin, China; ^2^ Department of Endocrinology, First Medical Center of Chinese People’s Liberation Army (PLA) General Hospital, Beijing, China; ^3^ Department of Endocrinology, Beijing Chao-Yang Hospital, Capital Medical University, Beijing, China; ^4^ Graduate School, Chinese People’s Liberation Army (PLA) General Hospital, Beijing, China; ^5^ Department of Medical Oncology, Senior Department of Oncology, The Fifth Medical Center of Chinese People’s Liberation Army (PLA) General Hospital, Beijing, China; ^6^ The Second Medical Center of Chinese People’s Liberation Army (PLA) General Hospital, Beijing, China; ^7^ Department of Endocrinology, Eighth Medical Center of People’s Liberation Army (PLA) General Hospital, Beijing, China; ^8^ Shanghai National Research Centre for Endocrine and Metabolic Diseases, State Key Laboratory of Medical Genomics, Shanghai Institute for Endocrine and Metabolic Diseases, Ruijin Hospital, Shanghai Jiaotong University School of Medicine, Shanghai, China; ^9^ Department of Endocrinology, Dalian Central Hospital, Dalian, China; ^10^ Department of Endocrinology, First Hospital of Lanzhou University, Lanzhou, China; ^11^ Department of Endocrinology, Zhongshan University Sun Yat-sen Memorial Hospital, Guangzhou, China; ^12^ Department of Endocrinology, Southwest Medical University Affiliated Hospital, Luzhou, China; ^13^ First Affiliated Hospital of Guangxi Medical University, Nanning, China; ^14^ First Affiliated Hospital of Zhengzhou University, Zhengzhou, China; ^15^ Wuhan Union Hospital, Huazhong University of Science and Technology, Wuhan, China

**Keywords:** visceral adiposity, visceral adiposity index, Chinese visceral adiposity index, obesity, hypertension, prehypertension

## Abstract

**Purpose:**

The optimal adiposity index that is strongly associated with hypertension or prehypertension remains inconclusive in Chinese population. This study aimed to investigate the relationship between Chinese visceral adiposity index (CVAI) and hypertension and prehypertension, as well as to compare the discriminative power of CVAI, visceral adiposity index (VAI), body mass index (BMI), waist circumference (WC), waist to height ratio (WHtR), low-density lipoprotein cholesterol (LDL-C), and waist-to-hip ratio (WHR) with hypertension and prehypertension in Chinese general population.

**Patients and Methods:**

A total of 34732 participants from REACTION study were recruited. Multiple logistic regression analyses were performed to detect the association between adiposity indices (CVAI, VAI, BMI, WC, WHtR, WHR, LDL-C) and hypertension and prehypertension.

**Results:**

Multivariate logistic regression analysis showed that compared with other obesity indices, CVAI remained significantly associated with hypertension and prehypertension (Hypertension: odds ratio (OR) 3.475, 95% confidence interval (CI) 3.158-3.824, p<0.001 in total subjects; OR 2.762, 95% CI 2.369–3.221, p<0.001 in men; OR 3.935, 95% CI 3.465–4.469, p<0.001 in women, Prehypertension: OR 2.747, 95% CI 2.460-3.068, p<0.001 in total subjects; OR 2.605, 95% CI 2.176–3.119, p<0.001 in men; OR 2.854, 95% CI 2.465–3.304, p<0.001 in women).In a stratified analysis, CVAI was significantly associated with hypertension and prehypertension at any level of blood glucose, age or estimated glomerular filtration rate(eGFR).

**Conclusion:**

CVAI is significantly associated with hypertension and prehypertension. CVAI shows the superior discriminative ability for hypertension and prehypertension compared with VAI, BMI, WC, WHtR, WHR and LDL-C in Chinese general population.

## Introduction

Hypertension is a widespread disease with a lifetime prevalence of more than 90 percent.(1)In recent years, hypertension has been claimed to be the leading cause of all-cause mortality worldwide (1). Prehypertension, a high-risk cause of hypertension, is a prevalent condition affecting 25% to 50% of the population in both developed and developing countries (2). The Framingham Heart Study revealed that prehypertension usually progresses to hypertension within four years in the elderly population (3) and has been shown to raise the risk of myocardial infarction (MI) by 3.5 times and coronary heart disease (CHD) by 1.7 times (4).It is noted that 23.3 percent of Chinese individuals have hypertension and 41.3 percent have prehypertension (5). Nonetheless, in comparison to developed countries, the knowledge, treatment and control of hypertension in China remains low (6).

In recent years, increasing evidence suggested that visceral fat, rather than subcutaneous or total fat, was associated with elevated blood pressure (7–9). The gold standard for measuring fat deposition is computed tomography (CT) and magnetic resonance imaging (MRI) (10), but they are time-consuming and expensive for large epidemiological studies. Thus, the obesity index associated with hypertension and prehypertension remains significant. However, simple anthropometric measures were insufficient for evaluating the relationship between local obesity and the risk of hypertension or prehypertension (11), because these indices, such as body mass index (BMI) and waist circumference (WC) could not accurately evaluate visceral fat tissue. Although the visceral adiposity index (VAI) could provide information about visceral fat tissue function (12), some studies have revealed that VAI is not the most associated adiposity index with hypertension among Chinese (13, 14). Evaluating the predictive performance of VAI, which was established for Caucasians, on patients with metabolic illnesses in the Chinese community could result in erroneous results (15).

In comparison to Caucasians, Asians exhibit higher levels of body adiposity at lower BMI levels (14, 16), and are more likely to accumulate visceral fat (14, 17).Xia et al. established the Chinese visceral adiposity index (CVAI) using multivariate linear regression analysis in 485 people recruited from Xiamen, China in 2016, and validated it in 6495 subjects recruited from Shanghai, China (14). CVAI has been confirmed more associated with diabetes (15, 18), diabetes complications (19), atherosclerotic disease (20, 21), polycystic ovary syndrome (22), and subclinical hypothyroidism (23) than other adiposity indices in the Chinese population, while other studies have found a significant association between CVAI and diabetes in the Japanese population (24). Studies on the relationship between CVAI and hypertension are limited (25), and the relationship between CVAI and prehypertension has not been investigated. Besides, previous research has revealed an association between serum low-density lipoprotein cholesterol (LDL-C) levels and visceral fat as measured by MRI (10),nevertheless, it is unknown whether LDL-C is a more reliable predictor of hypertension or prehypertension than other visceral obesity indices.

The optimal adiposity index that is strongly associated with hypertension or prehypertension remains debatable and inconclusive among the Chinese population. Thus, the purpose of this study is to investigate the relationship between CVAI and hypertension and prehypertension, as well as to compare the discriminative power of CVAI, VAI, BMI, WC, waist to height ratio (WHtR), LDL-C, and Waist-to-hip ratio (WHR) with hypertension and prehypertension in elderly people in seven Chinese provinces.

## Material and Methods

### Participants and Study Design

A total of 47808 participants from the REACTION study (26) were investigated, they took part in the survey between March and December 2012, the participants were recruited from 7 regional centers:(Gansu, Guangdong, Henan, Hubei, Liaoning, Shanghai, and Sichuan).

The research program was authorized by the Human Research of Rui-Jin Hospital affiliated with the School of Medicine of Shanghai Jiao Tong University. Before collecting the data, all participants provided a written informed consent.

Subjects who received medications (hypotensive drugs, lipid-lowering drugs or drugs treating kidney diseases), who had missing important information or abnormal important information and with various kidney diseases were excluded. Finally, a total of 34732 subjects were included, of whom 10494 were male and 24238 were female.

### Medical History

The past medical history, current medication situation, alcohol consumption, smoking history, physical activity (information on physical activity was gathered using the short form of the International Physical Activity Questionnaire, high level of physical activity was defined as strenuous physical activity lasting more than 10 minutes at a time in the past seven days, strenuous activity refers to activities that make people breathing difficult, such as playing basketball, swimming, running; moderate level of physical activity was defined as did at least 10 minutes of physical activity in the past 7 days that requires people to breathe slightly harder than usual, such as jogging, playing table tennis, golf, or tai chi without strenuous physical activity; low level of physical activity was defined as not reporting any activity or reporting some activity, such as walking, but not being satisfied with the above moderate and high levels of physical activity), marital status and other basic information were collected using the standardized self-administered questionnaires.

### Physical Examination and Laboratory Measurements

The height, weight, WC, and hip circumference (HC) were measured and recorded. The systolic blood pressure (SBP) and diastolic blood pressure (DBP) were measured by the same tester 3 times, 5 minutes apart. Averages of three values of SBP and DBP were used for data analysis. The blood samples were drawn in the morning. All subjects fasted for 8-10 hours before the test. After fasting blood glucose (FBG) was extracted, subjects without or with diabetes were tested for 75 g oral glucose tolerance or 100 g steamed-bread meal, respectively, 2 hours post-load blood glucose (PBG) was extracted again 2 hours after the first blood drawing. Serum triglycerides (TG), LDL-C, high density lipoprotein cholesterol (HDL-C), total cholesterol(TC), aspartate transferase (AST), alanine transferase (ALT), glutamine transferase (GGT), serum creatinine (SCr), and other biochemical indexes were measured on an autoanalyzer (c16000 system, ARCHITECT ci16200 analyzer; Abbott Laboratories, Lake Bluff, Illinois, America); hemoglobin A1c (HbA1c) was measured by high-performance liquid chromatography method (Variant II and D-10 Systems; Bio-Rad, Hercules, California, America).

### Calculation and Definition of Variables

BMI was calculated by dividing the weight in kilograms by the height in meters squared (kg/m (2)). The estimated glomerular filtration rate (eGFR) was expressed in mL/min per 1.73 m(2) using the formula, eGFR = 175 × (SCr in mg/dL) −1.154 × age−0.203 × (0.742 for women) × (1.212 if African American). WHR was calculated as WC divided by HC. WHtR was calculated as WC divided by height. The VAI and CVAI were calculated as follows:

Males: VAI= [WC/(39.68 + (1.88×BMI)] ×(TG/1.03) ×(1.31/HDL)

CVAI = −267.93 + 0.68 × age(y)+ 0.03 × BMI (kg/m(2)) + 4.00 × WC (cm) + 22.00 × LgTG (mmol/L) − 16.32 × HDL-C (mmol/L)

Females: VAI= [WC/(36.58+(1.89×BMI)] ×(TG/0.81) ×(1.52/HDL).

CVAI = −187.32 + 1.71 × age(y)+ 4.32 × BMI (kg/m(2)) + 1.12 × WC (cm) + 39.76 × LgTG (mmol/L) − 11.66 × HDL-C (mmol/L).

We only tested blood pressure by measuring blood pressure since we eliminated patients who were taking drugs that could affect their blood pressure (BP). Hypertension was defined as SBP ≥ 140 mmHg or DBP ≥ 90 mmHg, prehypertension was defined as SBP 120-140 mmHg or DBP 80-90 mmHg.

CVAI and VAI were divided into four groups: the < 25% group (the control group), the 25–50% group, the 50–75% group, and the ≥ 75% group, according to quartile division of the subjects. BMI was divided into three groups: normal: <24 kg/m2 (the control group), overweight:24-27.9 kg/m(2), obesity: ≥28 kg/m(2). WC was divided into two groups: normal:<90cm for men or <85cm for women (the control group), high: ≥90cm for men or ≥85cm for women, WHR: normal:<0.90 for men or <0.85 for women (the control group), high: ≥0.90 for men or ≥0.85 for women. WHtR: normal:<0.50, high: ≥0.50.LDL-C: ideal < 2.6 mmol/L (the control group), appropriate:2.6-3.4, borderline high: 3.4–4.1 mmol/L, high: ≥ 4.1 mmol/L.

### Statistical Analysis

SPSS 24.0 (IBM, Chicago, Illinois) was used for the statistical analysis. Continuous variables were expressed as mean ± SD. Categorical variables were presented numerically (proportionally). One-way analysis of variance (ANOVA) was used to test the difference of continuous variables. Categorical variables were analyzed by chi-square test. Three multivariate logistic regression models were built to identify the associations between the adiposity indices and hypertension or prehypertension. Model 0 was unadjusted. Model 1 was adjusted for center, age and sex. Model 2 was additionally adjusted for previously diagnosed cardiovascular disease (CVD), previously diagnosed diabetes, current smoking status, current drinking status, marital status, physical activity. Model 3 was additionally adjusted for eGFR, HbA1C, GGT, AST ALT, TG, TC, HDL-C and SCr. The correlation between the adiposity indices and hypertension or prehypertension in subgroups of sex, age, blood glucose, and eGFR were investigated. The stratification analysis was performed for each subgroup.

All statistical tests were two sided, and P < 0.05 was considered statistically significant.

## Results

The study included 34,732 participants (**Table 1**), with 11621 (33.5%) having hypertension and 13,164(57.0%) having pre-hypertension among the 23,111 non-hypertensive participants. Hypertensive participants were older, had higher obesity indices such as CVAI, VAI, BMI, WC, WHR, and WHtR, had greater LDL-C, TC, TG, and lower HDL-C concentrations, had a higher percentage of smokers or drinkers, and had more previous CVD or diabetes than non-hypertensive participants. Compared with participants with ideal blood pressure, participants with prehypertension were older, had higher levels of obesity indices, had higher levels of LDL-C, TC and TG and lower levels of HDL-C, were more likely to smoke or drink frequently, and were more likely to have prior CVD or diabetes.

**Table 1:** Characteristics of study population by hypertension or pre hypertension.

	All subjects			No hypertension subjects		
	Hypertension	No hypertension	Pvalue	Prehypertension	Ideal BP	Pvalue
N	11621	23111		13164	9947	
Age, years	61 (55,68)	56 (51,61)	<0.001	57 (53,63)	54 (49,59)	<0.001
Sex, (%)			<0.001			<0.001
Male	4059 (34.9)	6435 (27.8)		4099 (31.1)	2336 (23.5)	
Female	7562 (65.1)	16676 (72.2)		9065 (68.9)	7611 (76.5)	
CVAI	122.76 (98.49,147.00)	95.46 (71.28,120.92)	<0.001	104.35 (81.22,129.07)	83.39 (60.72,107.46)	<0.001
VAI	2.05 (1.32,3.24)	1.74 (1.12,2.77)	<0.001	1.88 (1.20,2.97)	1.57 (1.04,2.48)	<0.001
BMI, Kg/m^2^	25.5 (23.4,27.8)	23.7 (21.7,25.9)	<0.001	24.3 (22.3,26.6)	22.9 (21.0,25.0)	<0.001
WC	90 (83,96)	84 (78,90)	<0.001	86 (92,80)	81 (75,88)	<0.001
WHR	0.91 (0.86,0.95)	0.88 (0.83,0.92)	<0.001	0.89 (0.84,0.93)	0.86 (0.82,0.91)	<0.001
WHtR	0.56 (0.52,0.60)	0.52 (0.49,0.56)	<0.001	0.54 (0.50,0.57)	0.51 (0.47,0.55)	<0.001
LDL-C, mmol/L	3.06 (2.46,3.67)	2.88 (2.32,3.48)	<0.001	2.94 (2.39,3.55)	2.79 (2.23,3.38)	<0.001
HDL-C, mmol/L	1.27 (1.08,1.49)	1.30 (1.09,1.53)	<0.001	1.28 (1.09,1.51)	1.32 (1.11,1.57)	<0.001
TC, mmol/L	5.21 (4.47,5.94)	4.97 (4.26,5.70)	<0.001	5.06 (4.33,5.79)	4.85 (4.17,5.58)	<0.001
TG, mmol/L	1.51 (1.08,2.17)	1.30 (0.94,1.87)	<0.001	1.38 (1.00,2.00)	1.20 (0.88,1.70)	<0.001
HbA1c, %	6.0 (5.7,6.4)	5.8 (5.6,6.2)	<0.001	5.9 (5.6,6.2)	5.8 (5.5,6.1)	<0.001
ALT, U/L	16 (12,23)	14 (11,20)	<0.001	15 (11,21)	14 (10,19)	<0.001
AST, U/L	21 (18,26)	20 (17,24)	<0.001	20 (17,25)	20 (16,24)	<0.001
GGT, U/L	23 (17,36)	19 (14,29)	<0.001	21 (15,31)	18 (13,27)	<0.001
eGFR, mL/ (min·1.73 m^2^)	87.94 (77.51,99.15)	90.71 (81.42,101.32)	<0.001	90.22 (80.93,100.74)	91.53 (82.09,102.05)	<0.001
Drinking, (%)			0.001			0.001
Never	8821 (75.9)	17297 (74.8)		9840 (74.7)	7457 (75.0)	
Occasional	1788 (15.4)	4544 (19.7)		2874 (18.8)	2070 (20.8)	
Frequently	1012 (8.7)	1270 (5.5)		850 (6.5)	420 (4.2)	
Smoking, (%)			0.038			0.026
Never	10052 (86.5)	19783 (85.6)		11218 (85.2)	8565 (86.1)	
Occasional	241 (2.1)	537 (2.3)		296 (2.2)	241 (2.4)	
Frequently	1328 (11.4)	2791 (12.1)		1650 (12.5)	1141 (11.5)	
Diabetes, (%)			<0.001			<0.001
Yes	1712 (14.7)	1892 (8.2)		1294 (9.8)	598 (6.0)	
No	9909 (85.3)	21219 (91.8)		11870 (90.2)	9349 (94.0)	
Physical activity, (%)			<0.001			0.028
Low	9685 (83.3)	18150 (78.6)		10464 (79.4)	7689 (77.3)	
Moderate	1369 (11.8)	3512 (15.2)		1903 (14.5)	1609 (16.2)	
High	567 (4.9)	1449 (6.3)		791 (6.1)	652 (6.6)	
Married/cohabitating, (%)			<0.001			0.153
Yes	10061 (86.6)	20630 (89.3)		11753 (89.3)	8877 (89.3)	
No	1560 (13.4)	2481 (10.7)		1411 (10.7)	1070 (10.7)	
CVD, (%)			<0.001			<0.001
Yes	892 (7.68)	857 (3.71)		602 (4.57)	255 (2.56)	
No	10729 (92.32)	22254 (96.29)		12562 (95.43)	9692 (97.44)	

Data were mean ± SD or median (IQR) for skewed variables or numbers (proportions) for categorical variables.

CVAI chinese visceral adiposity index, VAI visceral adiposity index, BMI body mass index, WC waist circumference, WHtR waist to height ratio, WHR waist-to-hip ratio, HbA1c glycosylated hemoglobin, ALT alanine transferase, AST aspartate transferase, GGT gamma-glutamyl transferase, TG triglyceride, TC Total cholesterol, LDL-C low-density lipoprotein cholesterol, HDL-C high-density lipoprotein cholesterol, eGFR estimated glomerular filtration rate, CVD cardiovascular disease.

**Table 2** shows OR and 95% CI of hypertension with the groups of the seven obesity indices. After further adjustments in Model 3, the second, third and fourth quartiles of CVAI(second quartile: OR 1.768,95%CI 1.628-1.920,P< 0.001; third quartile: OR 2.523, 95%CI 2.314-2.750, P<0.001; fourth quartile: OR 3.475, 95%CI 3.158-3.824, P<0.001), the second, third and fourth quartiles of VAI(second quartile: OR 1.371,95%CI 1.267-1.84,P< 0.001; third quartile: OR 1.632, 95%CI 1.492-1.786, P< 0.001; fourth quartile: OR 1.895, 95%CI 1.685-2.133, P<0.001), LDL - C at 2.6 to 3.4 mmol/L(OR 1.102,95% CI 1.019-1.191,P=0.015), LDL - C at 3.4 to 4.1 mmol/L(OR 1.376,95% CI 1.229-1.540, P< 0.001),LDL-C ≥ 4.1 mmol/L(OR 1.548,95% CI 1.315-1.821, P<0.001);BMI at 24-27.9 kg/m(2)(OR 1.755,95% CI 1.660-1.856, P<0.001), BMI ≥28 kg/m2(OR 2.562,95% CI 2.378-2.761, P< 0.001);WHtR≥0.5(OR 1.855,95% CI 1.739-1.979, P<0.001); WC≥90cm for men or ≥85cm for women(OR 1.653,95% CI 1.569-1.741, P<0.001);WHR ≥0.90 for men or ≥0.85 for women(OR 1.536,95% CI 1.431-1.648, P<0.001) remained significantly associated with hypertension.

**Table 2:** Association of CVAI, VAI, BMI, WC, WHtR, WHR and LDL-C with hypertension and prehypertension.

	Hypertension				Pre-hypertension			
Model	Model 0	Model 1	Model 2	Model 3	Model 0	Model 1	Model 2	Model 3
Variable	OR (95% CI)	OR (95% CI)	OR (95% CI)	OR (95% CI)	OR (95% CI)	OR (95% CI)	OR (95% CI)	OR (95% CI)
**CVAI**								
Q1	1	1	1	1	1	1	1	1
Q2	2.186(2.025,2.359)***	1.811(1.673,1.960)***	1.807(1.669,1.956)***	1.768(1.628,1.920)***	1.896(1.771,2.029)***	1.705(1.589,1.829)***	1.703(1.587,1.828)***	1.613(1.497,1.739)***
Q3	3.765(3.497,4.053)***	2.672(2.470,2.889)***	2.648(2.448,2.865)***	2.523(2.314,2.750)***	2.990(2.778,3.218)***	2.469(2.282,2.671)***	2.464(2.277,2.666)***	2.233(2.044,2.440)***
Q4	6.840(6.357,7.360)***	3.850(3.548,4.178)***	3.782(3.483,4.106)***	3.475(3.158,3.824)***	4.452(4.089,4.848)***	3.214(2.924,3.533)***	3.185(2.896,3.503)***	2.747(2.460,3.068)***
**VAI**								
Q1	1	1	1	1	1	1	1	1
Q2	1.287(1.747,1.986)***	1.408(1.311,1.512)***	1.416(1.318,1.521)***	1.371(1.267,1.484)***	1.227(1.143,1.317)***	1.358(1.260,1.464)***	1.365(1.266,1.471)***	1.327(1.220,1.444)***
Q3	1.562(1.464,1.667)***	1.751(1.631,1.880)***	1.752(1.631,1.882)***	1.632(1.492,1.786)***	1.595(1.483,1.715)***	1.823(1.686,1.970)***	1.829(1.692,1.977)***	1.702(1.539,1.882)***
Q4	1.863(1.747,1.986)***	2.247(2.092,2.413)***	2.227(2.072,2.393)***	1.895(1.685,2.133)***	1.899(1.761,2.047)***	2.247(2.072,2.435)***	2.246(2.070,2.436)***	1.889(1.645,2.168)***
**LDL,mmol/L**								
Ideal level	1	1	1	1	1	1	1	1
Appropriate level	1.182(1.120,1.374)***	1.003(0.945,1.064)	1.015(0.956,1.077)	1.102(1.019,1.191)*	1.278(1.202,1.358)***	1.133(1.062,1.209)***	1.138(1.066,1.214)***	1.173(1.075,1.280)**
Borderline high	1.521(1.428,1.619)***	1.189(1.109,1.274)***	1.216(1.133,1.304)***	1.376(1.229,1.540)***	1.407(1.306,1.517)***	1.165(1.075,1.263)***	1.182(1.090,1.282)***	1.219(1.069,1.390)**
High	1.863(1.747,1.986)***	1.272(1.167,1.385)***	1.297(1.190,1.414)***	1.548(1.315,1.821)***	1.684(1.528,1.857)***	1.342(1.208,1.490)***	1.359(1.223,1.510)***	1.399(1.153,1.697)***
**BMI, Kg/m^2^**								
Normal	1	1	1	1	1	1	1	1
Overweight	2.145(2.039,2.255)***	1.918(1.817,2.024)***	1.897(1.797,2.002)***	1.755(1.660,1.856)***	1.928(1.821,2.042)***	1.806(1.701,1.916)***	1.799(1.695,1.909)***	1.642(1.544,1.747)***
Obesity	3.587(3.358,3.832)***	2.913(2.712,3.129)***	2.857(2.659,3.070)***	2.562(2.378,2.761)***	3.002(2.726,3.307)***	2.649(2.396,2.930)***	2.620(2.369(2.898)***	2.300(2.073,2.551)***
**WHtR**								
Normal	1	1	1	1	1	1	1	1
High	2.973(2.804,3.153)***	2.140(2.010,2.277)***	2.101(1.974,2.237)***	1.855(1.739,1.979)***	1.528(1.457,1.603)***	1.459(1.389,1.533)***	1.461(1.390,1.535)***	1.365(1.296,1.438)***
**WC**								
Normal	1	1	1	1	1	1	1	1
High	2.541(2.427,2.662)***	1.857(1.766,1.952)***	1.829(1.739,1.924)***	1.653(1.569,1.741)***	2.210(2.093,2.334)***	1.855(1.751,1.964)***	1.842(1.739,1.951)***	1.656(1.560,1.758)***
**WHR**								
Normal	1	1	1	1	1	1	1	1
High	1.743(1.642,1.850)***	1.772(1.655,1.898)***	1.735(1.619,1.859)***	1.536(1.431,1.648)***	1.465(1.378,1.557)***	1.629(1.519,1.746)***	1.612(1.503,1.728)***	1.413(1.315,1.518)***

Model 0 was unadjusted. Model 1 was adjusted for center, age and sex. Model 2 was additionally adjusted for previously diagnosed CVD, previously diagnosed diabetes, current smoking status, current drinking status, marital status, physical activity. Model 3 was additionally adjusted for eGFR, HbA1C, GGT, AST ALT, TG, TC, HDL-C and SCr.

***P <0.001, **P <0.01, *P <0.05.

CVAI chinese visceral adiposity index, VAI visceral adiposity index, BMI body mass index, WC waist circumference, WHtR waist to height ratio, WHR waist-to-hip ratio, HbA1c glycosylated hemoglobin, ALT alanine transferase, AST aspartate transferase, GGT gamma-glutamyl transferase, TG triglyceride, TC Total cholesterol, LDL-C low-density lipoprotein cholesterol, HDL-C high-density lipoprotein cholesterol, eGFR estimated glomerular filtration rate, CVD cardiovascular disease, SCr creatinine.

**Table 2** shows OR and 95% CI of prehypertension with the groups of the seven obesity indices. After further adjustments in Model 3, the second, third and fourth quartiles of CVAI(second quartile: OR 1.613,95%CI 1.497-1.739,P< 0.001; third quartile: OR 2.233, 95%CI 2.044-2.440, P< 0.001; fourth quartile: OR 2.747, 95%CI 2.460-3.068, P<0.001), the second, third and fourth quartiles of VAI(second quartile: OR 1.327,95%CI 1.220-1.444,P< 0.001; third quartile: OR 1.702, 95%CI 1.539-1.882, P< 0.001; fourth quartile: OR 1.889, 95%CI 1.645-2.168, P<0.001), LDL - C at 2.6 to 3.4 mmol/L(OR 1.173,95% CI 1.075-1.280,P=0.001), LDL - C at 3.4 to 4.1 mmol/L(OR1.219,95%CI 1.069-1.390, P=0.003),LDL-C≥4.1 mmol/L(OR 1.399,95% CI 1.153-1.697, P< 0.001);BMI at 24-27.9 kg/m(2)(OR 1.642,95% CI 1.544-1.747, P< 0.001), BMI ≥28 kg/m(2)(OR 2.300,95% CI 2.073-2.551, P<0.001);WHtR≥0.5(OR 1.365,95% CI 1.296-1.438, P<0.001); WC≥90cm for men or ≥85cm for women(OR 1.656,95% CI 1.560-1.758, P<0.001);WHR ≥0.90 for men or ≥0.85 for women(OR 1.413,95% CI 1.315-1.518, P< 0.001) remained significantly associated with prehypertension.

Stratified analyses were conducted to verify the stability of the results. **Tables 3**–**6** show the results of stratified analysis by sex, blood glucose, age, and eGFR, in that order. Compared with participants with lower CVAI levels, subjects with higher CVAI levels (the third and fourth quartile) were significantly associated with hypertension and prehypertension in both genders and at any level of blood glucose, age or eGFR.

**Table 3:** Association of the CVAI, VAI, BMI, WC, WHtR, WHR, LDL-C index with hypertension and prehypertension by sex.

	Hypertension				Pre-hypertension			
Gender	Male		Female		Male		Female	
Variable	OR (95% CI)	P value	OR (95% CI)	P value	OR (95% CI)	P value	OR (95% CI)	P value
**CVAI**								
Q1	1		1		1		1	
Q2	1.441 (1.237,1.679)	<0.001	1.916 (1.734,2.116)	<0.001	1.622 (1.389,1.894)	<0.001	1.610 (1.476,1.756)	<0.001
Q3	2.043 (1.760,2.371)	<0.001	2.758 (2.474,3.037)	<0.001	1.802 (1.532,2.119)	<0.001	2.436 (2.185,2.716)	<0.001
Q4	2.762 (2.369,3.221)	<0.001	3.935 (3.465,4.469)	<0.001	2.605 (2.176,3.119)	<0.001	2.854 (2.465,3.304)	<0.001
**VAI**								
Q1	1		1		1		1	
Q2	1.351 (1.190,1.535)	<0.001	1.387 (1.252,1.536)	<0.001	1.408 (1.205,1.646)	<0.001	1.299 (1.174,1.437)	<0.001
Q3	1.486 (1.281,1.724)	<0.001	1.690 (1.506,1.897)	<0.001	1.678 (1.393,2.021)	<0.001	1.692 (1.500,1.908)	<0.001
Q4	1.720 (1.402,2.110)	<0.001	1.933 (1.668,2.239)	<0.001	1.814 (1.392,2.364)	<0.001	1.851 (1.572,2.180)	<0.001
**LDL,mmol/L**								
Ideal level	1		1		1		1	
Appropriate level	1.102 (0.964,1.259)	0.154	1.099 (0.998,1.211)	0.056	1.043 (0.884,1.231)	0.619	1.226 (1.106,1.358)	<0.001
Borderline high	1.389 (1.137,1.697)	0.001	1.366 (1.191,1.568)	<0.001	1.020 (0.788,1.320)	0.882	1.296 (1.112,1.510)	0.001
High	1.451 (1.080,1.948)	0.013	1.575 (1.293,1.917)	<0.001	1.171 (0.793,1.729)	0.429	1.498 (1.196,1.875)	<0.001
**BMI, Kg/m^2^**								
Normal	1		1		1		1	
Overweight	1.993 (1.805,2.201)	<0.001	1.639 (1.531,1.755)	<0.001	1.767 (1.567,1.993)	<0.001	1.592 (1.481,1.712)	<0.001
Obesity	2.751 (2.400,3.153)	<0.001	2.461 (2.250,2.693)	<0.001	2.569 (2.086,3.163)	<0.001	2.209 (1.959,2.492)	<0.001
**WHtR**								
Normal	1		1		1		1	
High	1.295 (1.179,1.422)	<0.001	1.811 (1.670,1.964)	<0.001	1.292 (1.177,1.420)	<0.001	1.393 (1.309,1.483)	<0.001
**WC**								
Normal	1		1		1		1	
High	1.749 (1.596,1.917)	<0.001	1.587 (1.488,1.692)	<0.001	1.546 (1.375,1.740)	<0.001	1.668 (1.574,1.810)	<0.001
**WHR**								
Normal	1		1		1		1	
High	1.461 (1.333,1.601)	<0.001	1.692 (1.510,1.895)	<0.001	1.369 (1.224,1.530)	<0.001	1.459 (1.328,1.604)	<0.001

Adjusted for center, age, previously diagnosed CVD, previously diagnosed diabetes, current smoking status, current drinking status, marital status, physical activity, eGFR, HbA1C, GGT, AST ALT, TG, TC, HDL-C and SCr.

CVAI chinese visceral adiposity index, VAI visceral adiposity index, BMI body mass index, WC waist circumference, WHtR waist to height ratio, WHR waist-to-hip ratio, HbA1c glycosylated hemoglobin, ALT alanine transferase, AST aspartate transferase, GGT gamma-glutamyl transferase, TG triglyceride, TC Total cholesterol, LDL-C low-density lipoprotein cholesterol, HDL-C high-density lipoprotein cholesterol, eGFR estimated glomerular filtration rate, CVD cardiovascular disease, SCr creatinine.

**Table 4:** Association of CVAI, VAI, BMI, WC, WHtR, WHR and LDL-C with hypertension and prehypertension by blood glucose.

	Hypertension						Pre-hypertension							
glucose,mmol/L	FBG < 6.1 and PBG < 7.8		6.11≤FBG<7.0 or 7.8≤PBG<11.1		FBG≥7.0 or PBG ≥11.1		FBG < 6.1 and PBG < 7.8		6.11≤FBG<7.0 or 7.8≤PBG<11.1		FBG≥7.0 or PBG ≥11.1			
Variable	OR (95% CI)	P value	OR (95% CI)	P value	OR (95% CI)	P value	OR (95% CI)	P value	OR (95% CI)	P value			OR (95% CI)	P value
**CVAI**														
Q1	1		1		1		1		1			1		
Q2	1.706(1.503,1.936)	<0.001	1.700(1.503,1.922)	<0.001	1.578(1.211,2.057)	0.001	1.648(1.487,1.827)	<0.001	1.466(1.298,1.655)	<0.001		1.433(1.070,1.920)		0.016
Q3	2.259(1.959,2.606)	<0.001	2.289(2.019,2.595)	<0.001	2.535(1.959,3.280)	<0.001	2.307(2.021,2.632)	<0.001	1.944(1.698,2.227)	<0.001		1.958(1.437,2.670)		<0.001
Q4	3.561(3.017,4.202)	<0.001	3.162(2.758,3.625)	<0.001	2.715(2.075,3.552)	<0.001	2.513(2.104,3.000)	<0.001	2.526(2.145,2.973)	<0.001		2.835(2.006,4.006)		<0.001
**VAI**														
Q1	1		1		1		1		1			1		
Q2	1.230(1.081,1.399)	<0.001	1.344(1.198,1.508)	<0.001	1.559(1.237,1.963)	<0.001	1.279(1.136,1.439)	<0.001	1.332(1.163,1.526)	<0.001		1.061(0.771,1.461)		0.134
Q3	1.335(1.140,1.564)	<0.001	1.600(1.408,1.818)	<0.001	1.720(1.343,2.203)	<0.001	1.530(1.313,1.782)	<0.001	1.718(1.471,2.008)	<0.001		1.453(1.024,2.063)		0.037
Q4	1.787(1.431,2.231)	0.002	1.710(1.451,2.015)	<0.001	1.699(1.258,2.294)	0.001	1.658(1.316,2.088)	<0.001	1.855(1.515,2.270)	<0.001		1.396(0.903,2.158)		0.715
**LDL,mmol/L**														
Ideal level	1		1		1		1		1			1		
Appropriate level	1.087(0.947,1.248)	0.237	1.157(1.038,1.290)	0.008	0.903(0.735,1.110)	0.331	1.162(1.023,1.320)	0.021	1.213(1.063,1.385)	0.004		1.011(0.749,1.366)		0.942
Borderline high	1.314(1.072,1.611)	0.008	1.406(1.203,1.643)	<0.001	1.200(0.893,1.612)	0.226	1.180(0.972,1.432)	0.095	1.277(1.049,1.555)	0.015		0.981(0.622,1.547)		0.934
High	1.375(1.021,1.851)	0.036	1.571(1.256,1.964)	<0.001	1.540(1.009,2.352)	0.046	1.401(1.051,1.868)	0.021	1.470(1.101,1.961)	0.009		0.973(0.501,1.890)		0.935
**BMI, Kg/m^2^**														
Normal	1		1		1		1		1			1		
Overweight	1.787(1.623,1.967)	<0.001	1.632(1.508,1.765)	<0.001	1.734(1.493,2.015)	<0.001	1.713(1.565,1.876)	<0.001	1.495(1.361,1.642)	<0.001		1.604(1.301,1.977)		<0.001
Obesity	2.949(2.558,3.400)	<0.001	2.298(2.077,2.543)	<0.001	2.048(1.698,2.470)	<0.001	2.118(1.785,2.514)	<0.001	2.186(1.887,2.533)	<0.001		2.446(1.793,3.336)		<0.001
**WHtR**														
Normal	1		1		1		1		1			1		
High	1.793(1.615,1.992)	<0.001	1.717(1.564,1.885)	<0.001	1.990(1.644,2.409)	<0.001	1.457(1.350,1.572)	<0.001	1.254(1.158,1.357)	<0.001		1.067(0.900,1.266)		0.454
**WC**														
Normal	1		1		1		1		1			1		
High	1.726(1.575,1.891)	<0.001	1.561(1.452,1.679)	<0.001	1.483(1.291,1.705)	<0.001	1.589(1.372,1.684)	<0.001	1.655(1.512,1.812)	<0.001		1.578(1.296,1.920)		<0.001
**WHR**														
Normal	1		1		1		1		1			1		
High	1.537(1.361,1.735)	<0.001	1.473(1.334,1.626)	<0.001	1.517(1.252,1.839)	<0.001	1.436(1.297,1.591)	<0.001	1.391(1.243,1.557)	<0.001		1.219(0.946,1.571)		0.125

Adjusted for center, age, sex, previously diagnosed CVD, previously diagnosed diabetes, current smoking status, current drinking status, marital status, physical activity, eGFR, GGT, AST ALT, TG, TC, HDL-C and SCr.

CVAI chinese visceral adiposity index, VAI visceral adiposity index, BMI body mass index, WC waist circumference, WHtR waist to height ratio, WHR waist-to-hip ratio, HbA1c glycosylated hemoglobin, ALT alanine transferase, AST aspartate transferase, GGT gamma-glutamyl transferase, TG triglyceride, TC Total cholesterol, LDL-C low-density lipoprotein cholesterol, HDL-C high-density lipoprotein cholesterol, eGFR estimated glomerular filtration rate, CVD cardiovascular disease, SCr creatinine.

**Table 5:** Association of CVAI, VAI, BMI, WC, WHtR, WHR and LDL-C with hypertension and prehypertension by age.

	Hypertension						Pre-hypertension					
age,year	age<55		55≤age<65		age≥65		age<55		55≤age<65		age≥65	
Variable	OR (95% CI)	P value	OR (95% CI)	P value	OR (95% CI)	P value	OR (95% CI)	P value	OR (95% CI)	P value	OR (95% CI)	P value
**CVAI**												
Q1	1		1		1		1		1		1	
Q2	2.160 (1.909,2.445)	<0.001	1.639 (1.439,1.867)	<0.001	1.307 (1.033,1.654)	0.026	1.679 (1.517,1.859)	<0.001	1.701 (1.503,1.924)	<0.001	1.308 (0.987,1.732)	0.061
Q3	3.325 (2.888,3.827)	<0.001	2.386 (2.089,2.725)	<0.001	1.738 (1.387,2.178)	<0.001	2.430 (2.126,2.778)	<0.001	2.317 (2.018,2.661)	<0.001	1.848 (1.398,2.442)	<0.001
Q4	4.658 (3.929,5.523)	<0.001	3.328 (2.878,3.849)	<0.001	2.564 (2.036,3.227)	<0.001	2.997 (2.464,3.596)	<0.001	3.158 (2.659,3.752)	<0.001	2.068 (1.542,2.771)	<0.001
**VAI**												
Q1	1		1		1		1		1		1	
Q2	1.438 (1.251,1.652)	<0.001	1.342 (1.189,1.515)	<0.001	1.319 (1.124,1.548)	0.001	1.371 (1.216,1.547)	<0.001	1.362 (1.188,1.562)	<0.001	1.137 (0.897,1.442)	0.289
Q3	1.796 (1.533,2.104)	<0.001	1.541 (1.344,1.768)	<0.001	1.585 (1.314,1.911)	<0.001	1.892 (1.637,2.187)	<0.001	1.592 (1.354,1.873)	<0.001	1.437 (1.073,1.925)	0.015
Q4	2.309 (1.888,2.825)	<0.001	1.778 (1.485,2.219)	<0.001	1.683 (1.307,2.167)	<0.001	2.024 (1.663,2.464)	<0.001	2.037 (1.625,2.554)	<0.001	1.130 (0.753,1.696)	0.556
**LDL,mmol/L**												
Ideal level	1		1		1		1		1		1	
Appropriate level	1.101 (0.959,1.266)	0.173	1.082 (0.961,1.219)	0.191	1.062 (0.908,1.242)	0.451	1.203 (1.060,1.365)	0.004	1.211 (1.054,1.392)	0.007	0.961 (0.754,1.224)	0.748
Borderline high	1.365 (1.113,1.674)	0.003	1.320 (1.116,1.562)	0.001	1.409 (1.117,1.777)	0.004	1.225 (1.009,1.487)	0.04	1.320 (1.076,1.619)	0.008	0.966 (0.666,1.401)	0.856
High	1.687 (1.257,2.265)	<0.001	1.540 (1.212,1.956)	<0.001	1.288 (0.917,1.809)	0.144	1.418 (1.062,1.892)	0.018	1.492 (1.106,2.011)	0.009	1.195 (0.692,2.064)	0.522
**BMI, Kg/m^2^**												
Normal	1		1		1		1		1		1	
Overweight	1.998 (1.802,2.214)	<0.001	1.678 (1.542,1.825)	<0.001	1.588 (1.422,1.773)	<0.001	1.706 (1.559,1.867)	<0.001	1.623 (1.471,1.791)	<0.001	1.390 (1.173,1.647)	<0.001
Obesity	3.266 (2.863,3.725)	<0.001	2.349 (2.095,2.633)	<0.001	2.030 (1.750,2.355)	<0.001	2.648 (2.275,3.081)	<0.001	2.037 (1.722,2.409)	<0.001	1.896 (1.452,2.476)	<0.001
**WHtR**												
Normal	1		1		1		1		1		1	
High	2.042 (1.832,2.276)	<0.001	1.846 (1.675,2.035)	<0.001	1.651 (1.427,1.910)	<0.001	1.507 (1.394,1.629)	<0.001	1.349 (1.243,.464)	<0.001	0.993 (0.865,1.139)	0.915
**WC**												
Normal	1		1		1		1		1		1	
High	1.843 (1.678,2.024)	<0.001	1.621 (1.499,1.754)	<0.001	1.521 (1.367,1.691)	<0.001	1.769 (1.619,1.932)	<0.001	1.665 (1.514,1.831)	<0.001	1.421 (1.211,1.668)	<0.001
**WHR**												
Normal	1		1		1		1		1		1	
High	1.648 (1.457,1.864)	<0.001	1.570 (1.412,1.747)	<0.001	1.392 (1.203,1.612)	<0.001	1.467 (1.324,1.625)	<0.001	1.385 (1.234,1.555)	<0.001	1.411 (1.146,1.737)	0.001

Adjusted for center, sex, previously diagnosed CVD, previously diagnosed diabetes, current smoking status, current drinking status, marital status, physical activity, eGFR, HbA1C, GGT, AST ALT, TG, TC, HDL-C and SCr.

CVAI chinese visceral adiposity index, VAI visceral adiposity index, BMI body mass index, WC waist circumference, WHtR waist to height ratio, WHR waist-to-hip ratio, HbA1c glycosylated hemoglobin, ALT alanine transferase, AST aspartate transferase, GGT gamma-glutamyl transferase, TG triglyceride, TC Total cholesterol, LDL-C low-density lipoprotein cholesterol, HDL-C high-density lipoprotein cholesterol, eGFR estimated glomerular filtration rate, CVD cardiovascular disease, SCr creatinine.

**Table 6:** Association of CVAI, VAI, BMI, WC, WHtR, WHR and LDL-C with hypertension and prehypertension by eGFR.

	Hypertension								Pre-hypertension							
eGFR, ml/min per 1.73 m^2^	eGFR≥90			60≤eGFR<90			eGFR<60		eGFR≥90			60≤eGFR<90			eGFR<60	
Variable	OR (95% CI)		P value	OR (95% CI)	P value		OR (95% CI)	P value	OR (95% CI)		P value	OR (95% CI)	P value		OR (95% CI)	P value
**CVAI**																
Q1	1			1			1		1			1			1	
Q2	1.893 (1.690,2.120)		<0.001	1.665 (1.471,1.884)	<0.001		1.232 (0.662,2.293)	0.51	1.673 (1.510,1.853)		<0.001	1.546 (1.381,1.730)	<0.001		1.793 (0.874,3.681)	0.111
Q3	2.740 (2.431,3.089)		<0.001	2.332 (2.050,2.653)	<0.001		1.951 (1.060,3.590)	0.032	2.148 (1.902,2.427)		<0.001	2.340 (2.048,2.673)	<0.001		2.397 (1.093,5.260)	0.029
Q4	3.739 (3.272,4.272)		<0.001	3.221 (2.793,3.16)	<0.001		2.743 (1.425,5.278)	0.003	2.802 (2.404,3.267)		<0.001	2.719 (2.304,3.207)	<0.001		4.516 (1.779,11.465)	0.002
**VAI**																
Q1	1			1			1		1			1			1	
Q2	1.507 (1.349,1.683)		<0.001	1.276 (1.135,1.435)	<0.001		0.807 (0.479,1.359)	0.42	1.317 (1.174,1.478)		<0.001	1.312 (1.156,1.490)	<0.001		3.200 (1.542,6.638)	0.002
Q3	1.729 (1.518,1.969)		<0.001	1.571 (1.377,1.791)	<0.001		1.421 (0.810,2.490)	0.22	1.799 (1.564,2.068)		<0.001	1.605 (1.382,1.865)	<0.001		2.129 (0.938,4.833)	0.071
Q4	2.049 (1.720,2.441)		<0.001	1.798 (1.519,2.128)	<0.001		1.640 (0.810,3.321)	0.169	1.924 (1.582,2.340)		<0.001	1.848 (1.510,2.262)	<0.001		2.974 (1.010,8.752)	0.048
**LDL,mmol/L**																
Ideal level	1			1			1		1			1			1	
Appropriate level	1.111 (0.993,1.242)		0.066	1.117 (0.997,1.251)		0.056	0.908 (0.570,1.446)	0.684	1.158 (1.026,1.307)		0.017	1.167 (1.026,1.327)	0.019		1.744 (0.857,3.550)	0.125
Borderline high	1.357 (1.149,1.602)		<0.001	1.430 (1.218,1.678)		0.001	0.966 (0.505,1.847)	0.916	1.116 (0.925,1.347)		0.251	1.322 (1.095,1.596)	0.004		1.507 (0.526,4.320)	0.445
High	1.623 (1.277,2.064)		<0.001	1.507 (1.196,1.898)		0.001	1.309 (0.507,3.381)	0.578	1.319 (0.997,1.745)		0.053	1.518 (1.151,2.003)	0.003		1.608 (0.334,7.754)	0.554
**BMI, Kg/m^2^**																
Normal	1			1			1		1			1			1	
Overweight	1.875 (1.728,2.035)		<0.001	1.650 (1.525,1.786)		<0.001	1.726 (1.240,2.403)	0.001	1.630 (1.495,1.777)		<0.001	1.635 (1.494,1.790)	<0.001		2.587 (1.556,4.301)	<0.001
Obesity	2.814 (2.527,3.134)		<0.001	2.333 (2.095,2.599)		<0.001	2.389 (1.548,3.687)	<0.001	2.522 (2.183,2.913)		<0.001	2.054 (1.762,2.394)	<0.001		3.602 (1.695,7.652)	0.001
**WHtR**																
Normal	1			1			1		1			1			1	
High	1.941 (1.769,2.219)		<0.001	1.774 (1.616,1.947)		<0.001	1.832 (1.182,2.839)	0.007	1.392 (1.295,1.497)		<0.001	1.339 (1.241,1.445)	<0.001		1.463 (0.986,2.171)	0.058
**WC**																
Normal	1			1			1		1			1			1	
High	1.764 (1.635,1.903)		<0.001	1.566 (1.454,1.688)		<0.001	1.341 (0.979,1.837)	0.068	1.700 (1.563,1.848)	<0.001		1.617 (1.481,1.765)	<0.001		1.853 (1.165,2.947)	0.009
**WHR**																
Normal	1			1			1		1			1			1	
High	1.586 (1.439,1.748)	<0.001		1.463 (1.317,1.626)	<0.001		1.688 (1.073,2.653)	0.023	1.438 (1.305,1.585)	<0.001		1.399 (1.255,1.561)		<0.001	1.400 (0.790,2.484)	0.249

Adjusted for center, age, sex, previously diagnosed CVD, previously diagnosed diabetes, current smoking status, current drinking status, marital status, physical activity, HbA1C, GGT, AST ALT, TG, TC, HDL-C and SCr.

CVAI chinese visceral adiposity index, VAI visceral adiposity index, BMI body mass index, WC waist circumference, WHtR waist to height ratio, WHR waist-to-hip ratio, HbA1c glycosylated hemoglobin, ALT alanine transferase, AST aspartate transferase, GGT gamma-glutamyl transferase, TG triglyceride, TC Total cholesterol, LDL-C low-density lipoprotein cholesterol, HDL-C high-density lipoprotein cholesterol, eGFR estimated glomerular filtration rate, CVD cardiovascular disease, SCr creatinine.

Compared with participants with lower VAI levels, subjects with higher VAI levels (the third and fourth quartile) were significantly associated with hypertension in both genders and at any level of blood glucose, age, and when eGFR≥90 ml/min per 1.73 m(2) and eGFR at 60-90 ml/min per 1.73 m(2). Compared with participants with lower VAI levels, subjects with higher VAI levels (the third and fourth quartile) were significantly associated with prehypertension in both genders, in normal blood glucose (FBG < 6.1 mmol/L and PBG < 7.8 mmol/L), when prediabetes (6.11 mmol/L ≤FBG<7.0 mmol/L or 7.8 mmol/L ≤PBG<11.1 mmol/L), when eGFR≥90ml/min per 1.73 m(2), eGFR at 60-90 ml/min per 1.73 m(2), age<55 years old and age at 55-65 years old.

People with appropriate level of LDL-C (2.6-3.4 mmol/L) were associated with hypertension when prediabetes. People with borderline high levels of LDL-C (3.4–4.1 mmol/L) were associated with hypertension in both genders, in all age subgroups, in normal blood glucose, when prediabetes, when eGFR≥90ml/min per 1.73 m(2) and eGFR at 60-90 ml/min per 1.73 m(2). People with appropriate level of LDL-C were associated with prehypertension in female, in normal blood glucose, when prediabetes, when age<55 years old, age at 55-65 years old, eGFR≥90ml/min per 1.73 m(2) and eGFR at 60-90 ml/min per 1.73 m(2). People with borderline high levels of LDL-C were associated with prehypertension in female, when prediabetes, when age<55 years old, age at 55-65 years old and eGFR at 60-90 ml/min per 1.73 m(2).

People who are overweight (BMI at 24-27.9 kg/m(2)) or obesity (BMI ≥28 kg/m(2)) were significantly associated with hypertension and prehypertension in both genders and at any level of blood glucose, age and eGFR.

People with high WHtR (WHtR ≥ 0.5) were associated with hypertension in both genders and at any level of blood glucose, age and eGFR. People with high WHtR were associated with prehypertension in both genders, in normal blood glucose, when prediabetes, when age<55 years old, age at 55-65 years old, eGFR≥90ml/min per 1.73 m(2) and eGFR at 60-90 ml/min per 1.73 m(2).

People with high WC (WC≥90cm for men or ≥85cm for women) were associated with hypertension in both genders and at any level of blood glucose, age and when eGFR≥90ml/min per 1.73 m(2) and eGFR at 60-90 ml/min per 1.73 m(2). People with high WC were associated with prehypertension in both genders and at any level of blood glucose, age and eGFR.

People with high WHR (WHR ≥0.90 for men or ≥0.85 for women) were significantly associated with hypertension in both genders and at any level of blood glucose, age and eGFR. People with high WHR were significantly associated with prehypertension in both genders, in all age subgroups, in normal blood glucose, when prediabetes, when eGFR≥90ml/min per 1.73 m(2) and eGFR at 60-90 ml/min per 1.73 m(2).

## Discussion

To the best of our knowledge, this is the first large-scale, multicenter study to investigate the relationship between CVAI and hypertension and prehypertension in a Chinese general population. The main findings of this study suggested that CVAI was significantly associated with hypertension and prehypertension in both men and women, even after adjusting for a wide spectrum of biochemical and lifestyle risk factors. VAI, BMI, WC, LDL-C, WHtR and WHR were also associated with hypertension and prehypertension but were inferior to CVAI. Further stratification indicated that CVAI was still significantly associated with hypertension and prehypertension at any level of blood glucose, age and eGFR.

In the present study, the performance of traditional obesity indices (BMI, WC, LDL-C, WHtR and WHR) were not as strong as CVAI in the association with hypertension and prehypertension. We consider the reasons are as follows: (1) BMI could not distinguish fat mass from lean mass and could not reflect fat distribution (27), and it may overestimate the risk of obesity related disorders in people with heavy muscle mass (13). (2) The indices of central obesity (WC, WHR and WHtR) incorporate both the abdominal subcutaneous and visceral depots (28), but the correlation between these indices and subcutaneous fat were stronger than that between these indices and visceral fat (17). (3) CVAI is an indicator that includes both lipid and obesity measures, but LDL-C is simply a type of blood fat. Assmann et al. also have proven that the number of clinical events is still alarming regardless of currently desirable LDL-C lowering therapies (29). We speculate that this is also the reason why LDL-C is not as good as CVAI in the present study. Generally speaking, the traditional obesity indices described above all have the same flaw in that they do not differentiate between visceral and subcutaneous fat tissue.

Evidences suggested that abdominal fat deposition were more strongly associated with cardiometabolic risk factors and chronic disease risk (30). The Dallas Heart Study evaluated fat tissue using an MRI scanner and discovered that visceral fat, but not total or subcutaneous fat, was significantly associated with incident hypertension (9). The Framingham Heart Study demonstrated that both computed tomography (CT) estimated visceral and abdominal subcutaneous adipose volumes were connected with hypertension in Caucasian participants, with the former having a higher effect (7). A cross-sectional study of 11529 middle-aged Chinese population demonstrated that visceral fat was closely associated with the risk of hypertension and prehypertension (31).

VAI (32) and CVAI (14)were designed to predict visceral fat tissue in European and Chinese populations respectively. In this study, VAI was also associated with hypertension and prehypertension, but was inferior to CVAI or even other traditional obesity indices. High VAI levels were found to be merely a weak risk factor for hypertension in an Iranian study (33), and a cross-sectional study of 14,573 participants in Jiangxi, China reported that VAI was inferior to traditional obesity indices in predicting hypertension (13), these two studies were consistent with our findings. Xia et al. discovered that VAI was poorly associated with adipose tissue area measured using CT in 485 Chinese participants (AUROC: 0.69[0.65–0.73], P < 0.001), while the association between CVAI and adipose tissue area was significant (AUROC: 0.83 [0.79–0.86], P < 0.001) (14). According to the 2002 Expert Consultation, Asian populations had a higher percentage of body fat than Caucasians of the same age, gender, and BMI (34). Previous research found that Asians in the New York area have a lower BMI but a larger percentage of visceral fat than age-matched Caucasians (16). Therefore, we consider that VAI is not suitable for Chinese population due to the race variations in visceral fat tissue, and the powerful predictive ability of CVAI for hypertension and prehypertension in this study indicates that it is an obesity index worth attention.

Among the various obesity indices (CVAI, VAI, WC, and BMI), Han et al. found that CVAI performed the best in predicting incident hypertension among 9359 people in rural Henan Province, China (25), whereas our study was large-scale and multicenter, and involved a community-based urban population. Therefore, the current study population is more representative than Han’s. Furthermore, Han et al. did not include WHR and WHtR in their analysis, so the current study’s content is more thorough. The findings of our study were essentially consistent with those of Han et al., and serve as a supplement to Han et al.’s study, with greater relevance for the population in various regions of China. Besides, a study of 91 adults with growth hormone deficiency in Chongqing found that CVAI exhibited the strongest associations with SBP and DBP, while VAI had similar but weaker associations (35). The subjects of our study were general population, but our findings were similar. This demonstrates that the applicability of CVAI as an indicator of obesity to Chinese people, whether healthy or ill.

As far as we know, this study is the first to report a relationship between CVAI and prehypertension, and our findings suggested that the correlation between CVAI and prehypertension is stronger than the traditional obesity indices and VAI. Prehypertension is usually closely correlated with target organ injury, for example early arteriosclerosis, small vascular injury, coronary artery calcification, vascular remodeling and left ventricular hypertrophy (4). Some people could have prehypertension without awareness or diagnosis of it over the years, Unfortunately, they might develop complications during this time even without symptoms. Therefore, we recommend that people with high CVAI values monitor their blood pressure frequently and change lifestyle early to avoid developing prehypertension, hypertension or complications.

CVAI was significantly associated with hypertension and prehypertension in both genders, but the OR value of women was higher than that of men, indicating that CVAI had a stronger ability to predict elevated blood pressure in women. Wakabayashi reported that obesity was more associated with prehypertension or hypertension in women than in men (36). It has been discovered that women in Asia tend to carry more fat around their waists than men (37) and there was a greater association between visceral adipose tissue and adverse metabolic outcomes in women than in men (38, 39). Researches in rodents have provided evidence of sex differences in the role of adipocyte-derived factors in aldosterone secretion, in obese female rats, leptin may contribute to increased blood pressure by stimulating secretion of aldosterone, but the importance of leptin-induced increases in aldosterone secretion in the setting of obesity in male rodents was not observed (40). Therefore, we advise that women may require female-specific and more aggressive therapeutic and lifestyle management for obesity and elevated blood pressure than men.

In the stratified analysis, CVAI was still significantly associated with hypertension and prehypertension at any level of blood glucose, age or eGFR. This implies that the correlation between CVAI and elevated blood pressure is very stable, and further suggests that CVAI can be a strong obesity index indicating elevated blood pressure in Chinese population. This is, to the best of our knowledge, the first stratified analysis of the relationship between CVAI and hypertension and prehypertension.

The following are some of the potential mechanisms linking visceral obesity to hypertension (41). Visceral fat contains more large adipocytes than subcutaneous fat, as adipocytes grow larger, they become dysfunctional, large adipocytes are insulin-resistant, whereas small adipocytes are more insulin-sensitive (42). Insulin resistance was linked to the pathogenesis of hypertension *via* endothelial dysfunction, sodium retention, increased sympathetic activity and vascular hypertrophy (11) (1). Adiponectin levels are lower in visceral fat (43), and a low serum level of adiponectin has been identified as an independent risk factor for the development of hypertension (44) (2). Visceral fat is associated with higher leptin levels (43), and plasma leptin concentration predicts the onset of hypertension independently (45) (3).Visceral fat tissue usually infiltrated with activated macrophages, leading in increased secretion of different proinflammatory cytokine (46), which are associated with elevated blood pressure (47) (4). Preliminary evidence suggests that activation of an adipose renin–angiotensin system is associated with high blood pressure in a visceral fat model (45).

The current study is the first to explore the association between CVAI and prehypertension, and studies on the relationship between CVAI and hypertension are limited, this is the first large-scale, multicenter study to investigate the relationship between CVAI and hypertension. Moreover, our research benefited from a vast collection of multiple community-based samples, and the distribution of various districts in China was broadly representative. Furthermore, in this study, confounding factors were controlled for as much as possible. Nevertheless, some limitations also exist. First, since the current study was cross-sectional, only associations rather than causal relationships were established. Second, in epidemic investigation, white coat hypertension was unavoidable.

## Conclusion

In conclusion, we found that high CVAI levels were significantly associated with hypertension and prehypertension in the Chinese general population. VAI, BMI, WC, LDL-C, WHtR and WHR were also associated with hypertension and prehypertension but were inferior to CVAI. Based on the findings, we recommend using CVAI in both genders in Chinese as the most dependable, applicative and inexpensive index for identifying high risk of hypertension and prehypertension, as well as facilitating critical steps to prevent or delay the occurrence of hypertension or prehypertension.

## Data Availability Statement

The datasets used to support this study are not freely available due to participants’ privacy protection.

## Ethics Statement

The study protocol was approved by the Committee on Human Research at Rui Jin Hospital affiliated with the School of Medicine, Shanghai Jiao Tong University. Informed consents were provided by all participants before data collection. The patients/participants provided their written informed consent to participate in this study.

## Author Contributions

BL and JW have contributed equally to this work and share first authorship. BL and JW contributed to the conception and design of the study. XZ, YL, WW, ZG, XT, LY, QW, ZL, GQ, LC and GN recruited the subjects and supervised the study. BL analyzed the data and wrote the initial draft of the paper. YM, BL, and JW contributed to the writing, reviewing, and revising of the manuscript. All authors contributed to the article and approved the submitted version.

## Funding

The study is supported by the Chinese Society of Endocrinology, the Key Laboratory for Endocrine and Metabolic Diseases of Ministry of Health (1994DP131044), the National Key New Drug Creation and Manufacturing Program of Ministry of Science and Technology (2012ZX09303006-001), the National High Technology Research and Development Program of China (863 Program, 2011AA020107), National Science Foundation of China (81300717), National Science and Technology Major Project 288 (2011ZX09307-001-08), the REACTION Study, Beijing Municipal Science and Technology Commission Project (Z201100005520014), Golden Seed Project of Beijing Chaoyang Hospital (No.CYJZ202135).

## Conflict of Interest

The authors declare that the research was conducted in the absence of any commercial or financial relationships that could be construed as a potential conflict of interest.

## Publisher’s Note

All claims expressed in this article are solely those of the authors and do not necessarily represent those of their affiliated organizations, or those of the publisher, the editors and the reviewers. Any product that may be evaluated in this article, or claim that may be made by its manufacturer, is not guaranteed or endorsed by the publisher.
